# Supplementary figures and images for: Prehabilitation to improve outcomes afteR Autologous sTem cEll transplantation (PIRATE): A pilot randomised controlled trial protocol
Source: PLoS One. 2023 Apr 27;18(4):e0277760. doi: 10.1371/journal.pone.0277760 (PMC10138261; doi:10.1371/journal.pone.0277760)

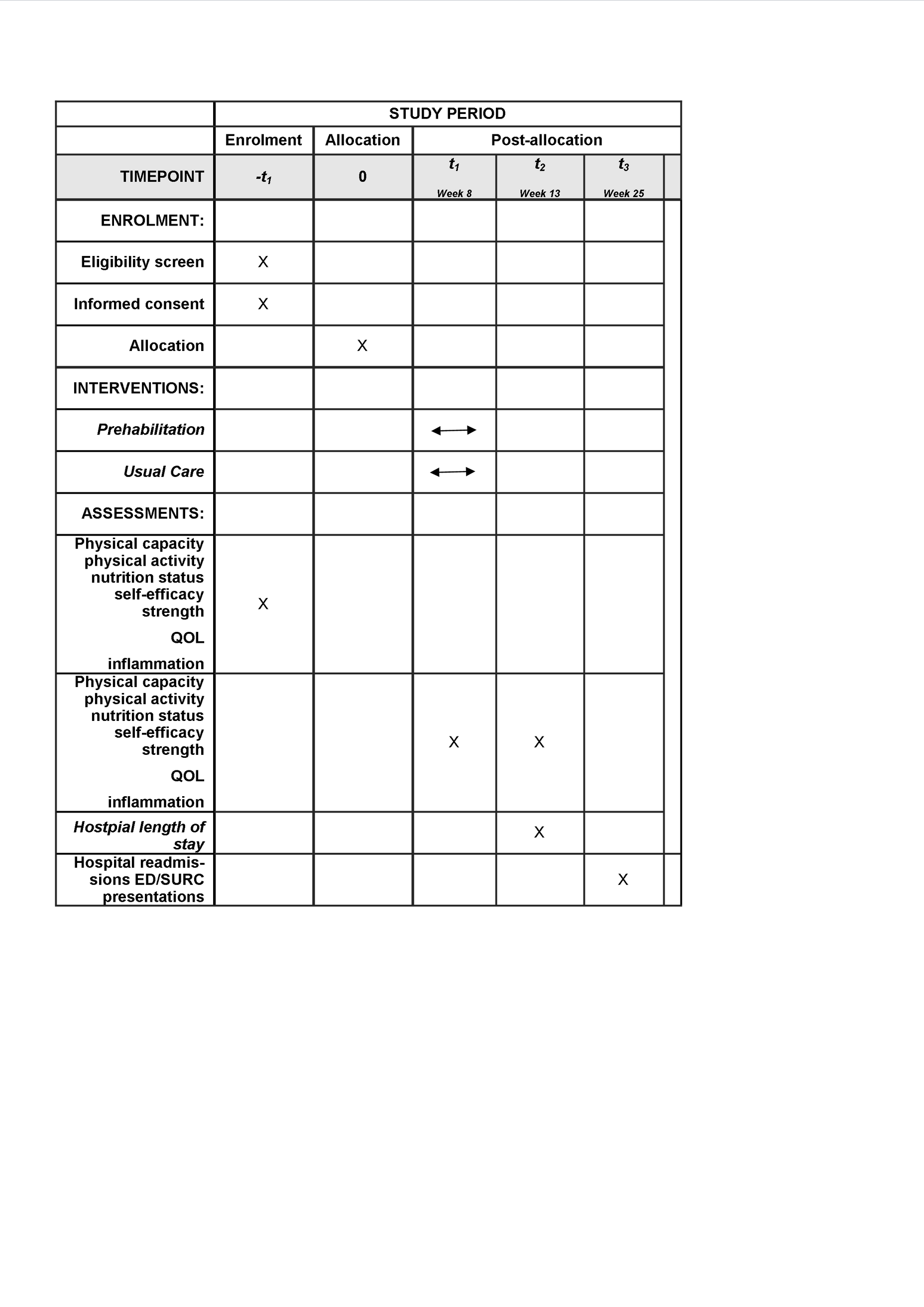

Supplement: S1 Fig — (TIF) [file pone.0277760.s002.tif]
