# Supplementary material for: Prehabilitation to improve outcomes afteR Autologous sTem cEll transplantation (PIRATE): A pilot randomised controlled trial protocol
Source: PLoS One. 2023 Apr 27;18(4):e0277760. doi: 10.1371/journal.pone.0277760 (PMC10138261; doi:10.1371/journal.pone.0277760)
Supplement: S1 File — This file is a questionnaire developed by the researchers that will be used to assess self-efficacy for physical activity using the Health Action Process Approach. (DOCX) [file pone.0277760.s003.docx]

Additional File Self-efficacy for Physical Activity Questionnaire

**Self-efficacy for physical activity**

Please answer the following statement in relation to the circumstances below.

Please tick the best answer for you.

Are you confident that you can permanently be regularly physically active?

| ***“I am confident that I can permanently be regularly physically active…”*** | | | | |
| --- | --- | --- | --- | --- |
| … even if I have side-effects (e.g. nausea) of the cancer therapy | Not at all ☐ | Partially agree ☐ | Mostly agree ☐ | Totally agree☐ |
| … even if I am tired | Not at all ☐ | Partially agree ☐ | Mostly agree ☐ | Totally agree☐ |
| … even if you have pain when exercising | Not at all ☐ | Partially agree ☐ | Mostly agree ☐ | Totally agree☐ |
| … even if you were too busy with other activities or appointments | Not at all ☐ | Partially agree ☐ | Mostly agree ☐ | Totally agree☐ |
| … even if you had to exercise alone | Not at all ☐ | Partially agree ☐ | Mostly agree ☐ | Totally agree☐ |
| … even if you felt stressed | Not at all ☐ | Partially agree ☐ | Mostly agree ☐ | Totally agree☐ |
| … even if you felt depressed | Not at all ☐ | Partially agree ☐ | Mostly agree ☐ | Totally agree☐ |
