# Supplementary material for: Prehabilitation to improve outcomes afteR Autologous sTem cEll transplantation (PIRATE): A pilot randomised controlled trial protocol
Source: PLoS One. 2023 Apr 27;18(4):e0277760. doi: 10.1371/journal.pone.0277760 (PMC10138261; doi:10.1371/journal.pone.0277760)
Supplement: S2 File — (DOCX) [file pone.0277760.s004.docx]

**Additional File 2. Rationale and psychometric properties of outcome measures**

*Measurement of primary outcome – physical capacity – 6-minute walk test*

The six minute walk test will be used to assess walking capacity. The 6-minute walk test is commonly used in trials of exercise after stem cell transplant [1]. The six minute walk is a valid and reliable measure of physical function in cancer survivors [2]. It will be completed according to published recommendations [3] with the exception of a practice test. Completion of the test without practice has demonstrated good reliability in other populations [4].

*Measurement of secondary outcomes*

*Objective physical activity*

Physical activity will be measured using a tri-axial accelerometer-based activity monitor (ActivPAL^TM^, PAL Technologies Ltd., Glasgow, UK). The ActivPAL^TM^ can record periods of time spent walking, standing sitting and walking, sit-to-stand transitions, step count and cadence over a maximum of 10 days. Physical activity will be expressed as the amount of moderate physical activity. This will be derived from the ActivPAL^TM^ data using a cut point of 100 steps per minute to define moderate intensity [5-7]. The amount of physical activity completed will be compared against guidelines that recommend at least 150 minutes of moderate intensity physical activity per week [8] and three times weekly moderate intensity exercise for 30 minutes per week [9]. The ActivPal^TM^ will also measure sedentary behaviour through time spent sitting and lying.

The ActivPAL^TM^ has been shown to provide valid estimates of energy expenditure and body transitions in non-hospitalised cancer survivors [10]. It has also demonstrated validity in a number of other populations including hospital inpatients,[11] community dwelling older adults,[12] and sedentary overweight adults [13]. The ActivPalTM has also demonstrated high compliance (98%) with continuous wear in people with cancer [14, 15]. Previous studies have recommended that monitoring is completed for 6 days or more [14] with continuous wear deemed more accurate for monitoring light and sedentary activity [16] .

*Inflammation*

Venous blood samples will be taken in the week of the patient’s assessment for the rehabilitation program and the week following completion of the rehabilitation program to determine the impact of physical activity levels on levels of systemic inflammation. An inverse relationship between inflammatory markers and physical activity levels (that is, lower levels of inflammation with higher volumes of physical activity) which can contribute cancer related symptoms including fatigue, mood and cognition as well as cardiovascular and cancer risk has been demonstrated previously [17-19]. It is hypothesised that the prehabilitation group will have higher levels of physical activity and thus lower levels of systemic inflammation.

*Health-Related Quality of Life*

Quality of life will be assessed using the European Organization for Research and Treatment of Cancer QoL Questionnaire-C30 (EORTC-QLQ C30). This is a multidimensional questionnaire comprising 30 items within different domains. It comprises of one global scale, five functional scales (physical, role, emotional, cognitive, and social), three symptom scales (fatigue, pain, and nausea) and six single item scales including dyspnoea, insomnia, appetite, constipation, diarrhoea and financial difficulties. This measure has demonstrated validity and reliability across a range of cancer settings [20] and is sensitive to change people undergoing chemotherapy [21]. The EORTC-QLQ C30 will be supplemented by the EORTC QLQ-HDC29 module which is specific to people undergoing high dose chemotherapy for stem cell transplant [22].

*Self-efficacy for physical activity*

Maintenance self-efficacy for physical activity will be measured using a 7-item questionnaire adapted from the Health Action Process Approach [23, 24]. Self-efficacy will be assessed as it is an important determinant of physical activity behaviour change [25]. Seven items will measure confidence adhering to regular physical activity. An example item is “I am confident that I can permanently be regularly physically active even if I have side-effects (e.g.,nausea) of the cancer-therapy” with a response format from 1“not at all” to 4 “totally agree.” A similar method has been used in a previous trial of a physical activity behavior change for cancer survivors [26].

*Nutritional status*

Nutritional status will be measured using the Patient-Generated Subjective Global Assessment (PG-SGA). The PG-SGA is a quick, easy to use tool comprising four self-reported items, weight, food intake, symptoms, activities and function and a physical exam to determine nutritional risk and deficit [27]. The PG-SGA is valid for use in people with cancer, [28] demonstrates high sensitivity (80%) and specificity (72%) in people receiving cancer treatment and predicts survival in people with haematological malignancies [27].

*Handgrip strength*

A handgrip dynamometer (Jamar®, Patterson Medical, IL, USA) will be used to assess upper limb muscle performance. It will be assessed using the best measure of 6 trials (3 in each hand). Participants will be asked to use the dynamometer at maximal effort in their each hand, alternating hands after each measure.[29] The handgrip dynamometer is a reliable outcome of strength that has also been able to predict all-cause mortality and functional decline [30, 31] in middle aged and older adults. Handgrip strength is also an indicator of nutrition status [32] which can predict cachexia in patients with cancer [33] and hospital costs [34].

**References**

*1. Liang Y, Zhou M, Wang F, Wu Z. Exercise for physical fitness, fatigue and quality of life of patients undergoing hematopoietic stem cell transplantation: a meta-analysis of randomized controlled trials. Jap J Clin Oncol. 2018;48(12):1046-57. Epub 2018/10/03. doi: 10.1093/jjco/hyy144. PubMed PMID: 30277514.*

*2. Schmidt K, Vogt L, Thiel C, Jäger E, Banzer W. Validity of the six-minute walk test in cancer patients. Int J Sports Med. 2013;34(7):631-6. Epub 2013/02/28. doi: 10.1055/s-0032-1323746. PubMed PMID: 23444095.*

*3. ATS statement: guidelines for the six-minute walk test. Am J Respir Crit Care Med. 2002;166(1):111-7. Epub 2002/07/02. doi: 10.1164/ajrccm.166.1.at1102. PubMed PMID: 12091180.*

*4. van Loo MA, Moseley AM, Bosman JM, de Bie RA, Hassett L. Test-re-test reliability of walking speed, step length and step width measurement after traumatic brain injury: a pilot study. Brain injury. 2004;18(10):1041-8. Epub 2004/09/17. doi: 10.1080/02699050410001672314. PubMed PMID: 15370902.*

*5. Dennett AM, Shields N, Peiris CL, Prendergast LA, O'Halloran PD, Parente P, et al. Motivational interviewing added to oncology rehabilitation did not improve moderate-intensity physical activity in cancer survivors: a randomised trial. J Physiother. 2018;64(4):255-63. Epub 2018/09/16. doi: 10.1016/j.jphys.2018.08.003. PubMed PMID: 30217412.*

*6. Rowe DA, Welk GJ, Heil DP, Mahar MT, Kemble CD, Calabro MA, et al. Stride rate recommendations for moderate-intensity walking. Med Sci Sports Exerc. 2011;43(2):312-8. Epub 2010/06/15. doi: 10.1249/MSS.0b013e3181e9d99a. PubMed PMID: 20543754.*

*7. Abel M, Hannon J, Mullineaux D, Beighle A. Determination of step rate thresholds corresponding to physical activity intensity classifications in adults. J Phys Act Health. 2011;8(1):45-51. Epub 2011/02/08. doi: 10.1123/jpah.8.1.45. PubMed PMID: 21297184.*

*8. Cormie P, Atkinson M, Bucci L, Cust A, Eakin E, Hayes S, et al. Clinical Oncology Society of Australia position statement on exercise in cancer care. Med J Aust. 2018;209(4):184-7. Epub 2018/05/03. PubMed PMID: 29719196.*

*9. Campbell KL, Winters-Stone KM, Wiskemann J, May AM, Schwartz AL, Courneya KS, et al. Exercise guidelines for cancer survivors: consensus statement from international multidisciplinary roundtable. 2019;51(11):2375-90. doi: 10.1249/mss.0000000000002116. PubMed PMID: 00005768-201911000-00023.*

*10. Skipworth RJ, Stene GB, Dahele M, Hendry PO, Small AC, Blum D, et al. Patient-focused endpoints in advanced cancer: criterion-based validation of accelerometer-based activity monitoring. Clin Nutr. 2011;30(6):812-21. Epub 2011/07/08. doi: 10.1016/j.clnu.2011.05.010. PubMed PMID: 21733604.*

*11. Taraldsen K, Askim T, Sletvold O, Einarsen EK, Bjastad KG, Indredavik B, et al. Evaluation of a body-worn sensor system to measure physical activity in older people with impaired function. Phys Ther. 2011;91(2):277-85. Epub 2011/01/08. doi: 10.2522/ptj.20100159. PubMed PMID: 21212377.*

*12. Grant PM, Dall PM, Mitchell SL, Granat MH. Activity-monitor accuracy in measuring step number and cadence in community-dwelling older adults. Journal of aging and physical activity. 2008;16(2):201-14. Epub 2008/05/17. doi: 10.1123/japa.16.2.201. PubMed PMID: 18483442.*

*13. Kozey-Keadle S, Libertine A, Lyden K, Staudenmayer J, Freedson PS. Validation of wearable monitors for assessing sedentary behavior. Med Sci Sports Exerc. 2011;43(8):1561-7. Epub 2011/01/15. doi: 10.1249/MSS.0b013e31820ce174. PubMed PMID: 21233777.*

*14. Maddocks M, Byrne A, Johnson CD, Wilson RH, Fearon KC, Wilcock A. Physical activity level as an outcome measure for use in cancer cachexia trials: a feasibility study. Support Care Cancer. 2010;18(12):1539-44. Epub 2009/12/04. doi: 10.1007/s00520-009-0776-2. PubMed PMID: 19956982.*

*15. Dennett AM, Peiris CL, Shields N, Prendergast LA, Taylor NF. Cancer survivors awaiting rehabilitation rarely meet recommended physical activity levels: an observational study. Rehabil Oncol. 2018;36(4):214-22. doi: 10.1097/01.reo.0000000000000132. PubMed PMID: 01893697-201810000-00007.*

*16. Mâsse LC, Fuemmeler BF, Anderson CB, Matthews CE, Trost SG, Catellier DJ, et al. Accelerometer data reduction: a comparison of four reduction algorithms on select outcome variables. Med Sci Sports Exerc. 2005;37(11 Suppl):S544-54. Epub 2005/11/19. doi: 10.1249/01.mss.0000185674.09066.8a. PubMed PMID: 16294117.*

*17. Gleeson M, Bishop NC, Stensel DJ, Lindley MR, Mastana SS, Nimmo MA. The anti-inflammatory effects of exercise: mechanisms and implications for the prevention and treatment of disease. Nat Rev Immunol. 2011;11(9):607-15. Epub 2011/08/06. doi: 10.1038/nri3041. PubMed PMID: 21818123.*

*18. Seruga B, Zhang H, Bernstein LJ, Tannock IF. Cytokines and their relationship to the symptoms and outcome of cancer. Nat Rev Cancer. 2008;8(11):887-99. Epub 2008/10/11. doi: 10.1038/nrc2507. PubMed PMID: 18846100.*

*19. Rogers LQ, Vicari S, Trammell R, Hopkins-Price P, Fogleman A, Spenner A, et al. Biobehavioral factors mediate exercise effects on fatigue in breast cancer survivors. Med Sci Sports Exerc. 2014;46(6):1077-88. Epub 2013/11/12. doi: 10.1249/mss.0000000000000210. PubMed PMID: 24212124; PubMed Central PMCID: PMCPmc4435796.*

*20. Luckett T, King MT, Butow PN, Oguchi M, Rankin N, Price MA, et al. Choosing between the EORTC QLQ-C30 and FACT-G for measuring health-related quality of life in cancer clinical research: issues, evidence and recommendations. Ann Oncol. 2011;22(10):2179-90. Epub 2011/02/23. doi: 10.1093/annonc/mdq721. PubMed PMID: 21339384.*

*21. Uwer L, Rotonda C, Guillemin F, Miny J, Kaminsky MC, Mercier M, et al. Responsiveness of EORTC QLQ-C30, QLQ-CR38 and FACT-C quality of life questionnaires in patients with colorectal cancer. Health Qual Life Outcomes. 2011;9:70. Epub 2011/08/24. doi: 10.1186/1477-7525-9-70. PubMed PMID: 21859485; PubMed Central PMCID: PMCPMC3170175.*

*22. Velikova G, Weis J, Hjermstad MJ, Kopp M, Morris P, Watson M, et al. The EORTC QLQ-HDC29: a supplementary module assessing the quality of life during and after high-dose chemotherapy and stem cell transplantation. Eur J Cancer. 2007;43(1):87-94. Epub 2006/11/04. doi: 10.1016/j.ejca.2006.09.004. PubMed PMID: 17081744.*

*23. Schwarzer R, Lippke S, Luszczynska A. Mechanisms of health behavior change in persons with chronic illness or disability: the Health Action Process Approach (HAPA). Rehabil Psychol. 2011;56(3):161-70. Epub 2011/07/20. doi: 10.1037/a0024509. PubMed PMID: 21767036.*

*24. Schwarzer R. Modeling Health Behavior Change: How to Predict and Modify the Adoption and Maintenance of Health Behaviors. 2008;57(1):1-29. doi: 10.1111/j.1464-0597.2007.00325.x.*

*25. McAuley E, Blissmer B. Self-efficacy determinants and consequences of physical activity. Exerc Sport Sci Rev. 2000;28(2):85-8. Epub 2000/07/21. PubMed PMID: 10902091.*

*26. Ungar N, Wiskemann J, Sieverding M. Physical activity enjoyment and self-efficacy as predictors of cancer patients' physical activity level. Frontiers Psychol. 2016;7:898. doi: 10.3389/fpsyg.2016.00898. PubMed PMID: PMC4914598.*

*27. Jager-Wittenaar H, Ottery FD. Assessing nutritional status in cancer: role of the Patient-Generated Subjective Global Assessment. Current opinion in clinical nutrition and metabolic care. 2017;20(5):322-9. Epub 2017/06/01. doi: 10.1097/mco.0000000000000389. PubMed PMID: 28562490.*

*28. Bauer J, Capra S, Ferguson M. Use of the scored Patient-Generated Subjective Global Assessment (PG-SGA) as a nutrition assessment tool in patients with cancer. European journal of clinical nutrition. 2002;56(8):779-85. Epub 2002/07/18. doi: 10.1038/sj.ejcn.1601412. PubMed PMID: 12122555.*

*29. Roberts HC, Denison HJ, Martin HJ, Patel HP, Syddall H, Cooper C, et al. A review of the measurement of grip strength in clinical and epidemiological studies: towards a standardised approach. Age Ageing. 2011;40(4):423-9. Epub 2011/06/01. doi: 10.1093/ageing/afr051. PubMed PMID: 21624928.*

*30. Bohannon RW. Dynamometer measurements of hand-grip strength predict multiple outcomes. Percept Mot Skills. 2001;93(2):323-8.*

*31. Sasaki H KF, Yamada M, Fujita S. Grip strength predicts cause-specific mortality in middle-aged and elderly persons. Am J Med 2007;120(4):337-42.*

*32. Guerra RS, Fonseca I, Pichel F, Restivo MT, Amaral TF. Handgrip strength cutoff values for undernutrition screening at hospital admission. Eur J Clinical Nutrit. 2014;68(12):1315-21. Epub 2014/10/30. doi: 10.1038/ejcn.2014.226. PubMed PMID: 25351643.*

*33. Ozorio GA, Barao K, Forones NM. Cachexia stage, patient-generated subjective global assessment, phase angle, and handgrip strength in patients with gastrointestinal cancer. Nutrition and Cancer. 2017;69(5):772-9. Epub 2017/05/20. doi: 10.1080/01635581.2017.1321130. PubMed PMID: 28524706.*

*34. Guerra RS, Amaral TF, Sousa AS, Pichel F, Restivo MT, Ferreira S, et al. Handgrip strength measurement as a predictor of hospitalization costs. Eur J Clin Nutrit. 2015;69(2):187-92. Epub 2014/11/06. doi: 10.1038/ejcn.2014.242. PubMed PMID: 25369830.*
