# Supplementary material for: Prehabilitation to improve outcomes afteR Autologous sTem cEll transplantation (PIRATE): A pilot randomised controlled trial protocol
Source: PLoS One. 2023 Apr 27;18(4):e0277760. doi: 10.1371/journal.pone.0277760 (PMC10138261; doi:10.1371/journal.pone.0277760)
Supplement: S1 Data — (DOCX) [file pone.0277760.s005.docx]

| **protocol** |
| --- |
| **Prehabilitation to Improve outcomes afteR Autologous sTem cEll transplantation (PIRATE) – A randomised controlled trial** |
| **Version: 3**  **Date: 4/2/2021** |
|  |
| **Author/s:**  **Amy Dennett, Nicholas Taylor, Judi Porter, Stephen Ting** |
| **Statement of Compliance**  This study will be conducted in compliance with all stipulation of this protocol, the conditions of the ethics committee approval, the NHMRC National Statement on ethical Conduct in Human Research (2007) and the Note for Guidance on Good Clinical Practice (CPMP/ICH-135/95). |

# **Table of Contents**

**Contents**

[**Table of Contents** 2](#_Toc14689304)

[**1.** **Glossary of Abbreviations & Terms** 5](#_Toc14689305)

[**2.** **Study Sites** 5](#_Toc14689306)

[2.1 Study Location/s 5](#_Toc14689307)

[**3.** **Introduction/Background Information** 5](#_Toc14689308)

[3.1 Lay Summary 5](#_Toc14689309)

[3.2 Introduction 6](#_Toc14689310)

[**4.** **Study Objectives** 7](#_Toc14689312)

[4.1 Study Aims 7](#_Toc14689313)

[4.2 Hypothesis……………………………………………………………………………………………………………………..8](#_Toc14689314)

[4.3 Data Collection tools](#_Toc14689314) 8

[**5.** **Study Design** 11](#_Toc14689315)

[5.1 Study Type & Design & Schedule 11](#_Toc14689316)

[**6.** **Study Population** 12](#_Toc14689318)

[6.1 Recruitment Procedure 12](#_Toc14689319)

[6.2 Inclusion Criteria 12](#_Toc14689320)

[6.3 Exclusion Criteria 12](#_Toc14689321)

[6.4 Consent 12](#_Toc14689322)

**7. Intervention details……………………………………………………………………………………………………….……13**

7.1 Intervention group: Prehabilitation…………………………………………………………………………………….13

7.2 Control group…………………..…………………………………………………………………………………………………13

[**8.** **Participant Safety and Withdrawal** 13](#_Toc14689323)15

[8.1 Risk Management and Safety 15](#_Toc14689324)

[8.2 Handling of Withdrawals 16](#_Toc14689325)

[8.3 Replacements 16](#_Toc14689326)

[**9.** **Statistical Methods** 16](#_Toc14689327)

[9.1 Sample Size Estimation & Justification 16](#_Toc14689328)

[9.2 Statistical Methods To Be Undertaken 16](#_Toc14689329)

[**10.** **Data Security & Handling** 17](#_Toc14689330)

[10.1 Details of where records will be kept & How long will they be stored 17](#_Toc14689331)

[10.2 Confidentiality and Security 17](#_Toc14689332)

10.3 Oversight and monitoring……………………………………………..………………………………….…………..17

[**10.** **Appendix** 18](#_Toc14689334)

[**11.** **References** 18](#_Toc14689335)

| **STUDY SYNOPSIS** | |  |  |
| --- | --- | --- | --- |
| Title: | Prehabilitation to improve outcomes after autologous stem cell transplantation (PIRATE) | | |
| Short Title: | Prehabilitation for AutoSCT | | |
| Design: | Randomised controlled trial | | |
| Study Centre: | Eastern Health – Box Hill Hospital | | |
| Study Aim: | Evaluate the effectiveness of exercise and nutrition prehabilitation on physical capcity | | |
| Inclusion Criteria: | - aged 18 years and over - a haematological malignancy and are waitlisted for autologous stem cell transplant - able to give written informed consent. | | |
| Exclusion Criteria: | - a medical condition that contraindicates participation in an exercise-based rehabilitation program - an Australian modified Karnofsky Performance status of <60 or ECOG >2 - cognitive impairment precluding informed consent | | |
| Number of Planned Subjects: | 22 | | |
| Statistical Methods: | - Descriptive statistics - Linear mixed models | | |
| Subgroups: | None | | |

## **Glossary of Abbreviations & Terms**

| **Abbreviation** | **Description (using lay language)** |
| --- | --- |
| Auto SCT | Autologous Stem Cell Transplant |
| EMR | Electronic Medical Record |
| SURC | Symptom and Urgent Review Clinic |
| AKPS | Australian-modified Karnofsky Performance Status |
| ECOG | Eastern Cooperative Oncology Group |
| PG-SGA | Patient-Generated Subjective Global Assessment |
| HR | Heart rate |
| RM | Repetition maximum |
| BMI | Body Mass Index |

## **Study Sites**

### Study Location/s

| **Site** | **Address** | **Contact Person** | **Phone** | **Email** |
| --- | --- | --- | --- | --- |
| Eastern Health: Box Hill Hospital | Clinical Research Office  Level 2, 5 Arnold St  Box Hill 3128 | Amy Dennett | 03 9095 2442 | [Amy.dennett@easternhealth.org.au](mailto:Amy.dennett@easternhealth.org.au) |

## **Introduction/Background Information**

### Lay Summary

People with blood cancers suffer extensive physical and psychological burden due to

intensive cancer treatment. Blood stem cell transplants are one form of treatment that improves survival of blood cancers such as multiple myeloma, leukaemia and lymphoma. However, this demanding treatment results in significant side-effects including reduced immune function, serious infections, sensation changes, fatigue and profound deconditioning. This treatment also creates significant emotional distress and can impact negatively on a patient’s quality of life. People receiving stem cell transplant often experience long recovery times and in some cases, long-term functional impairments.

Providing rehabilitation may help overcome these problems leading to faster recovery and better quality of life. Rehabilitation has been shown to be safe, reduce fatigue, and improve strength and quality of life in people being treated with stem cell transplant. However, while there are clear benefits to people participating in rehabilitation as part of their cancer treatment, the optimal timing of rehabilitation is unknown. Participating in rehabilitation prior to stem cell transplant may be particularly beneficial to build strength and fitness reserves to better cope with the procedure.

This project will find out if providing early rehabilitation (prehabilitation) prior to stem cell transplant builds physical strength and fitness to prevent problems after stem cell transplant, leading to faster recovery from treatment.

### Introduction

Autologous stem cell transplantation (Auto SCT) is an effective procedure that provides long-term disease control and is standard care for many haematological malignancies including multiple myeloma, lymphoma and leukaemia.^1^ In selected diseases, the survival rate of people receiving Auto SCT is up to 94% at 10 years.^2^ Auto SCT is increasingly available to people with haematological malignancies, with over 2,000 stem cell transplants completed in Australia annually,^3^ including approximately 30 per annum completed at Eastern Health.

While survival from Auto SCT is high, this procedure places patients at risk of significant short and long-term effects. People preparing for Auto SCT receive high-doses of chemotherapy and radiotherapy to eliminate tumor cells from the body. This results in a period of immunosuppression whereby people are at risk of severe infections, prolonged bed rest and immobility during a lengthy hospitalization whilst awaiting bone marrow stem cell recovery. This intensive treatment frequently results in a myriad of toxic effects including fatigue, peripheral neuropathy, deconditioning and emotional distress causing subsequent functional decline and poor quality of life.^4^ These problems may persist long after transplant with 35% of long-term transplant recipients experiencing fatigue.^5^ Persistent muscle weakness, pain, cognitive impairment and distress are also common.^6^ Autologous transplant recipients are also have shorter life expectancy and higher risk of secondary cancers than the general population and experience a high rate of hospital readmission.^2,7^

Exercise-based rehabilitation plays an important role in mitigating the negative effects of cancer and its treatment. Guidelines recommend people with cancer participate in thrice weekly aerobic and resistance exercise training to improve health outcomes.^8^ Exercise training reduces fatigue, improves strength and quality of life in people receiving Auto SCT.^9,10^ However, it is unknown how best to deliver exercise-based rehabilitation for people receiving Auto SCT. Supervised exercise yields the greatest benefits for cancer survivors.^8^ However, the majority of previous trials for people receiving Auto SCT has included unsupervised training.^9,10^ The optimal time period to provide exercise-based rehabilitation is also unknown, with most trials completed to date targeting the peri and post-transplant period.^9,10^ One trial found an 18 week supervised exercise-rehabilitation program after transplant was not cost-effective.^11^ Preliminary evidence suggests that training prior to Auto SCT may be superior to post-transplant.^10^

The addition of nutrition interventions to exercise may further enhance outcomes after autologous stem cell transplant as this group have complex nutrition needs and are at high risk of malnutrition.^12^ Nutrition interventions provided before and after autologous stem cell transplant reduce weight-loss and hospital length of stay.^13-16^

The period before Auto SCT may be a critical period to intervene with rehabilitation (prehabilitation). Prior to stem cell transplant, patients already experience impaired physical function and quality of life.^17^ Functional performance status is positively associated with survival after transplant^18^ therefore, patients preparing for transplant need to achieve a minimum level of function (KPS≥70 or ECOG ≤2).^1^ Prehabilitation aims to build functional reserve to prepare people to cope with the physical and psychological demands of treatment. Systematic reviews have shown prehabilitation to be effective in people with other cancers, for example those receiving surgery.^19^ However, limited high quality studies have been completed for people with haematological cancer. One non-randomised feasibility study including 29 participants has shown a 4 to 6 week supervised exercise-rehabilitation program was safe and well accepted by patients preparing for Auto SCT.^20^ Patients receiving prehabilitation required shorter length of stay (4 days) and earlier recovery of blood counts than the control group.^20^ Previous studies on prehabilitation for Auto SCT also only focused on exercise, and did not consider other interventions such as nutrition education. No trials have evaluated the impact of multidisciplinary prehabilitation for people preparing for Auto SCT.

*Rationale/Justification*

Prehabilitation has been likened to preparing for a marathon as it builds functional reserve

for major procedures. There is good evidence exercise and nutrition interventions can

manage debilitating treatment-related side-effects and improve function and quality of life of

cancer survivors. Prehabilitation is effective for improving outcomes after surgical

interventions for cancer, including reducing hospital length of stay and post-operative

complications. Therefore, prehabilitation may enhance recovery after Auto SCT, where debilitating side-effects are greater than for other cancers. Prehabilitation is not standard care for people with cancer and its effect on recovery after Auto SCT is largely unknown.

## **Study Objectives**

### Study Aims

This pragmatic, pilot randomised controlled trial will evaluate whether a multidisciplinary, exercise-based rehabilitation program offered up to 8 weeks before Auto SCT improves physical capacity after transplant.

Objectives:

1. Determine preliminary efficacy and safety of prehabilitation for Auto SCT. The primary outcome will be physical capacity at 4 weeks post-transplant. Secondary patient outcomes will include: adverse events, time to recovery of blood counts (haemoglobin, neutrophils and platelets), blood product transfusion requirements, inflammation, nutritional status, muscle strength, symptoms, physical activity levels, self-efficacy and quality of life.
2. Determine the impact of prehabilitation from a health service perspective in relation to hospital length of stay and hospital readmissions.

### Hypothesis

It is hypothesised that patients receiving prehabilitation for Auto SCT will experience less functional decline after Auto SCT, reduced complications and shorter inpatient length of stay. These results will inform the need for the implementation of prehabilitation in hospital settings for people undergoing Auto SCT.

### Data Collection Tools

Participants will complete an assessment of physical capacity, physical activity, inflammation, health-related quality of life, self-efficacy and nutritional status and strength at baseline and after the intervention phase at week 8 (pre-transplant) and week 13 (approximate, 1-month post-transplant infusion). A trained allied health clinician blind to group allocation will complete baseline and follow-up assessments to ensure blinding of outcome measures. Peripheral blood count analysis and blood product use will be completed by an independent assessor, blinded to group allocation. The assessments will take place at 4.1 Oncology/Haematology ward, Box Hill Hospital or in the patient’s home.

Health service data including hospital length of stay, emergency department and Symptom and Urgent Review Clinic (SURC) presentations, hospital readmissions will be obtained from Eastern Health databases. Time to recovery of haemoglobin, neutrophils and platelets will be calculated from routine data collected by the electronic medical record (EMR).

*Measurement of primary outcome – physical capacity – 6-minute walk test*

The six minute walk test will be used to assess walking capacity. The 6-minute walk test is commonly used in trials of exercise after stem cell transplant.^10^ The six minute walk is a valid and reliable measure of physical function in cancer survivors.^21^ It will be completed according to published recommendations^22^ with the exception of a practice test. Completion of the test without practice has demonstrated good reliability in other populations.^23^

*Measurement of secondary outcomes*

*Adverse events*

Adverse events related to the intervention as defined by the World Health Organization^24^ will be documented to report safety of the intervention. The event may or may not be related to the intervention, but it occurs while the person is participating in the intervention phase (during prehabilitation) of the trial. Adverse events will be categorised as minor adverse events or serious adverse events. A minor adverse event is defined as an incident that occurs while the person is participating in the intervention that results in no injury or minor injury (e.g. fatigue, exacerbation of pre-existing musculoskeletal pain) that requires none or minor medical intervention. A serious adverse event is defined as an incident that occurs while the person is participating in the intervention that results in death, serious injury or re-hospitalisation. Reasons for non-participation in an exercise session or non-completion of the program will be recorded (e.g. pain, fatigue, unwell). Complications related to the stem cell transplant procedure will also reported for each group (e.g. infection, bleeding, mucositis, parental nutrition requirements, intensive care support).

*Objective physical activity*

Physical activity will be measured using a tri-axial accelerometer-based activity monitor (ActivPAL^TM^, PAL Technologies Ltd., Glasgow, UK). The ActivPAL^TM^ can record periods of time spent walking, standing sitting and walking, sit-to-stand transitions, step count and cadence over a maximum of 10 days. Physical activity will be expressed as the amount of moderate physical activity. This will be derived from the ActivPAL^TM^ data using a cut point of 100 steps per minute to define moderate intensity.^25-27^ The amount of physical activity completed will be compared against guidelines that recommend at least 150 minutes of moderate intensity physical activity per week^28^ and three times weekly moderate intensity exercise for 30 minutes per week.^8^ The ActivPal^TM^ will also measure sedentary behaviour through time spent sitting and lying.

The ActivPAL^TM^ has been shown to provide valid estimates of energy expenditure and body transitions in non-hospitalised cancer survivors.^29^ It has also demonstrated validity in a number of other populations including hospital inpatients,^30^ community dwelling older adults,^31^ and sedentary overweight adults.^32^ The ActivPalTM has also demonstrated high compliance (98%) with continuous wear in people with cancer.^33,34^

Activity monitors will be placed on the anterior mid-thigh, inside a ziplock plastic sleeve and covered with an adhesive, water-proof dressing to allow continuous wear. Participants will be provided with written instructions on hygiene care and how to care for, remove and re-apply the activity monitor (for bathing and swimming) and asked to wear it for a period of 8 consecutive days. Participants will be instructed to continue with their usual level of activity. They will also be asked to keep an activity logbook (Appendix A) to supplement the activity monitor data. Previous studies have recommended that monitoring is completed for 6 days or more^33^ with continuous wear deemed more accurate for monitoring light and sedentary activity.^35^ Only complete 24 hour recording days will be included for analysis. However, as monitors may need to be removed for the purpose of swimming or bathing, evidence of non-wear matching with the activity logbook will still be included. Participants will be asked to wear the monitor for the whole period of data collection except in the case of complete water immersion (swimming and bathing). If the monitor needs to be removed for any reason, participants will be asked to place it back on as soon as possible and document the event.

*Inflammation*

Venous blood samples will be taken in the week of the patient’s assessment for the rehabilitation program and the week following completion of the rehabilitation program to determine the impact of physical activity levels on levels of systemic inflammation. Patients will be instructed not to undertake moderate to vigorous intensity exercise for 24 hours prior to collection due to the acute increase in inflammatory markers following a bout of exercise. If patients suffer from upper limb lymphoedema, samples will be taken from the unaffected arm for safety. Samples will be taken to measure C-reactive Protein using standard techniques. An inverse relationship between inflammatory markers and physical activity levels (that is, lower levels of inflammation with higher volumes of physical activity) which can contribute cancer related symptoms including fatigue, mood and cognition as well as cardiovascular and cancer risk has been demonstrated previously.^36-38^ It is hypothesised that the prehabilitation group will have higher levels of physical activity and thus lower levels of systemic inflammation. Samples will be taken by a qualified pathologist blinded to group assignment and assayed in a commercial Eastern Health laboratory.

*Health-Related Quality of Life*

Quality of life will be assessed using the European Organization for Research and Treatment of Cancer QoL Questionnaire-C30 (EORTC-QLQ C30, Appendix B). This is a multidimensional questionnaire comprising 30 items within different domains. It comprises of one global scale, five functional scales (physical, role, emotional, cognitive, and social), three symptom scales (fatigue, pain, and nausea) and six single item scales including dyspnoea, insomnia, appetite, constipation, diarrhoea and financial difficulties. This measure has demonstrated validity and reliability across a range of cancer settings^39^ and is sensitive to change people undergoing chemotherapy.^40^ The EORTC-QLQ C30 will be supplemented by the EORTC QLQ-HDC29 module (Appendix C) which is specific to people undergoing high dose chemotherapy for stem cell transplant.^41^

*Self-efficacy for physical activity*

Maintenance self-efficacy for physical activity will be measured using a 7 item questionnaire (Appendix D) adapted from the Health Action Process Approach.^42,43^ Seven items will measure confidence adhering to regular physical activity. An example item is “I am confident that I can permanently be regularly physically active even if I have side-effects (e.g.,nausea) of the cancer-therapy” with a response format from 1“not at all” to 4 “totally agree.” A similar method has been used in a previous trial of a physical activity behavior change for cancer survivors.^44^

*Nutritional status*

Nutritional status will be measured using the Patient-Generated Subjective Global Assessment (PG-SGA) (Appendix E). The PG-SGA is a quick, easy to use tool comprising four self-reported items, weight, food intake, symptoms, activities and function and a physical exam to determine nutritional risk and deficit.^45^ The PG-SGA is valid for use in people with cancer,^46^ demonstrates high sensitivity (80%) and specificity (72%) in people receiving cancer treatment and predicts survival in people with haematological malignancies.^45^

*Handgrip strength*

A handgrip dynamometer (Jamar®, Patterson Medical, IL, USA) will be used to assess upper limb muscle performance. It will be assessed using the best measure of 6 trials (3 in each hand). Participants will be asked to use the dynamometer at maximal effort in their each hand, alternating hands after each measure.^47^ The handgrip dynamometer is a reliable outcome of strength that has also been able to predict all-cause mortality and functional decline^48,49^ in middle aged and older adults. Handgrip strength is also an indicator of nutrition status^50^ which can predict cachexia in patients with cancer^51^ and hospital costs.^52^

*Time to stem cell engraftment*

Number of days from transplant to engraftment. After Auto SCT, patients are at higher risk anaemia, infection and bleeding until the stem cell graft has taken. Engraftment is defined as neutrophils >0.5 x 10^9^/L for three days without support and platelets >50 x 10^9^/L for five days without transfusion. Therefore, time taken to return to these values will be calculated from time of procedure to engraftment from routinely collected blood samples during a patient’s admission and/or clinic visits. These blood samples will be collected and assayed by a qualified pathologist blinded to group assignment in a commercial Eastern Health laboratory.

*Hospital length of stay*

Days that the patient is in the hospital from day of stem cell infusion to day of discharge. This will be collected from routinely collected hospital data.

*ED/SURC presentations*

Number of emergency department presentations and Symptom and Urgent Review Clinic (SURC) presentations over three months after discharge from the AutoSCT admission. These will be collected from routinely collected hospital data and the electronic medical record (EMR)

*Hospital re-admission*

Number of hospital readmissions over 3 months after discharge from the Auto SCT admission and associated inpatient days with each readmission. This will be collected from routinely collected hospital data.

Other routinely collected data will be used to describe the sample as follows: Age, gender, cancer type, cancer stage, treatment regime/s, co-morbidities, functional performance status (Australian Karnofsky Performance Status AKPS and ECOG), BMI.

# **Study Design**

### Study Type & Design

This study will be a single-blinded pilot randomised controlled trial (Figure 1).

## **Study Population**

### Recruitment Procedure

Eligible participants will be identified by stem cell transplant nurse consultants and haematologists at Eastern Health. Potential candidates will be advised about the study by transplant nurses and/or specialists verbally and/or through flyers (Appendix F). If a patient gives permission to being contacted about the research project, they will be contacted by a member of the research team who will provide them with details of the study and arrange to meet them at the haematology clinic or at home to provide them an opportunity to ask any questions and ask them to provide written informed consent. Each participant will retain a copy of their Participant Information and Consent Form. After obtaining consent and baseline measures, and they meet eligibility criteria, participants will be allocated to the prehabilitation group or control group according to an online computer generated randomisation program, [www.randomization.com](http://www.randomization.com) using permuted blocks. Assignments will be placed in sequentially numbered, opaque, sealed envelopes prior to study commencement by an independent researcher with no role in subject recruitment or administration of trial interventions.

### Inclusion Criteria

Participants recruited from haematology clinics at Eastern Health and affilitated haematology private practices where patients will be referred to Eastern Health for Auto SCT. Participants will be eligible if they: are aged 18 years and over; have a haematological malignancy and are waitlisted for autologous stem cell transplant; and are able to give written informed consent.

### Exclusion Criteria

Participants will be excluded if:

- A physiotherapist will screen for absolute exercise contra-indications in the medical record: unstable angina, arrhythmia, hypertension or heart failure, acute embolus/infarct, acute systemic infection with fever, dissecting aneurysm, acute myocarditis or pericarditis. The physiotherapist will obtain physician clearance to exercise if patient’s bloods and vital signs fall outside acceptable values (Appendix G). If patients have a medical condition that contraindicates participation in an exercise-based rehabilitation program as assessed by a physiotherapist or medical practitioner, they will be excluded (Appendix H).
- They have an Australian-modified Karnofsky Performance status of <60 or ECOG >2.
- They have a cognitive impairment that precludes their ability to provide written, informed consent as assessed by their treating clinician.

### Consent

A plain language statement will be provided at the beginning of the study and consent will be obtained prior to participation. Participants will have the opportunity to ask further questions and discuss the study with others, including their treating team, prior to providing consent.

# **Intervention details**

### INTERVENTION GROUP: PREHABILITATION

Participants randomized to the prehabilitation group will be offered a comprehensive physiotherapy assessment and twice weekly, 60-minute exercise classes for up to 8 weeks pre-transplant, supervised by a physiotherapist conducted at home. The exercise intervention will be in a circuit format and consist of aerobic and resistance exercise, completed at a moderate intensity (4-6 BORG rating of perceived exertion (RPE) and/or 60-80% Heart rate (HR) maximum for aerobic exercises, 60-80% 1-repetition maximum or 10-12 repetition maximum (RM) for resistance exercise in accordance with published guidelines.^8^ Exercise will be tailored to each patient following an assessment by the physiotherapist. Participants will complete a 5 minute warm up, aim to complete 20-25 minutes aerobic exercise, 20-25 minutes of resistance exercise, 5 minutes of flexibility or balance training (as indicated) and 5 minute cool down. Exercise intensity during aerobic exercise will be monitored by the physiotherapist using the modified BORG scale and using a portable heart rate monitor. During weeks one and two, participants will aim to work at a BORG RPE of 3 (moderate), and by week 8 participants will aim to work at a 5-6 (hard) on the scale. For resistance exercise, weights will be progressed once a participant is achieving 2 to 3 sets of 10-12 repetitions. Resistance exercise may include upper and lower body resistance exercise such as squats, step ups, free weights, wall push-ups, free weights, resistance exercise bands, Aerobic exercise may include walking, treadmill, stationary cycle/pedals,. Various upper and lower body stretches and balance exercise will also be incorporated into the program as required.

Participants in the prehabilitation group will be encouraged to complete an additional 30-minute aerobic exercise training session at home and will be provided with a Fitbit Inspire device and collaborate with the physiotherapist to set and review a daily steps goal to further assist compliance with exercise guidelines for people with cancer. The Fitbit Inspire is a device worn at the wrist that has the capability to collect data regarding physical activity, sleep and heart rate. The participant will be asked to wear the Fitbit device during waketime hours for the duration of the intervention period.

Data required for Fitbit set up: participant height, weight, age and gender; will be obtained from the most recent reading in the electronic medical record or in cases where these data are not available, will be measured during their first physiotherapy exercise session. The participant dashboard will be set-up with a specific trial log-in account on a device owned by Eastern Health. The participant will be provided with individual trial log-in details and instructed about the core functions on the Fitbit and Fitbit Dashboard on the chosen device by the supervising physiotherapist. The supervising physiotherapist will also provide education and technical support to improve compliance and safety as required throughout the intervention period. Administrator access to the Fitbit platform will be permitted for the research team. As a baseline, the Fitbit Inspire will be used to set a goal for and monitor daily steps and exercise minutes only.

The initial daily steps goal will be formulated and the participant will monitor their steps/day and exercise minutes progress throughout the intervention by looking at the Fitbit Inspire watch and/or the Fitbit dashboard using the account details provided by the research team.

Only data relating to the participant will be viewed and recorded. Data will be synced weekly with the Fitbit Dashboard and contents discussed within supervised exercise sessions. Data collected and recorded by the research team via the Fitbit Dashboard over the course of the intervention period will be steps/day, exercise minutes and hours worn– measured by record of valid heart rate data. Data will be downloaded in a de-identified form and stored in a password protected folder.

Written instructions with guidelines for exercise after cancer will also be given (Appendix I). Patients will be instructed to remain as active as possible and avoid prolonged periods of sitting and lying in the period following stem cell transplant.

Patients will also receive a comprehensive nutrition assessment with a dietitian via telephone or videoconference. The dietitian will provide written information and fortnightly phone calls in addition to the assessment over the 8 week (up to 4 sessions) period to patients offering tailored dietary education and advice based on their assessment results. They will also be referred to the standard sub-acute Oncology Rehabilitation Program at Wantirna Health for participation after study completion.

- 1. CONTROL GROUP

Participants randomized to the control group will receive a physiotherapy assessment and a standardized written handout with guidelines for exercise after cancer (Appendix I). They will also be referred to the standard sub-acute Oncology Rehabilitation Program at Wantirna Health for participation after study completion (Figure 1).

Recruitment: Eastern Health Haematology clinics

Measure: Physical capacity, physical activity level, nutrition status, self-efficacy, strength, health-related quality of life, inflammation

**Experimental n=11**

Multi-disciplinary prehabilitation

- Face to face physiotherapy assessment
- 2x weekly supervised exercise (16 sessions), 8 weeks
- Telehealth nutrition assessment
- 1x fortnightly nutrition phone call (4 sessions), 8 weeks
- Written handout on exercise and cancer

Measure: Physical capacity, physical activity level, nutrition status, self-efficacy, strength, health-related quality of life, inflammation

Measure: Physical capacity, physical activity level, nutrition status, self-efficacy, health-related quality of life, inflammation, time to blood count recovery, hospital length of stay

**Experimental n=11**

Usual inpatient/outpatient post-transplant care

**Control n=11**

Usual inpatient/outpatient post-transplant care

Time (weeks)

0

Approx 8

Auto Stem Cell Transplant admission

Approx 13

**Control n=11**

- Face to face physiotherapy assessment
- Written handout on exercise and cancer

Approx 25

Measure: readmissions, ED/SURC presentations

Figure 1. Trial design

1. **Participant Safety and Withdrawal**

## Risk Management and Safety

There are several potential benefits associated with this project. Participants may improve their physical fitness, function and emotional wellbeing. They may also find out more information about, and improve their physical activity levels and nutrition status. Information collated from this trial will assist clinicians working with people with cancer in designing appropriate prehabilitation programs to promote physical activity and nutrition and improve their function which has long term implications for patients through improved quality of life, prevention of tumour recurrence and improved life expectancy. There are also potential benefits for the health service as prehabilitation may be a cost-effective way to deliver supportive cancer care.

There are limited anticipated risks for patients participating in this project. There may be discomfort associated with having blood assays taken for the measures of inflammation. However, they will be taken by a qualified nurse or pathologist with standard procedure and collected simultaneously to other routine blood tests when able. Participants may also choose to opt out of giving blood samples and just complete the other assessments if they wish. Participants may find it uncomfortable answering questions related to their emotional well-being when completing the health-related quality of life questionnaires. Participants who indicate high levels of psychological distress, will be offered free counselling which can be facilitated by the researcher through their general practitioner or other appropriate support such as as Lifeline (13 11 14; www.lifeline.org.au), SANE Australia (1800 18 SANE (7263); www.sane.org.au) or Beyond Blue (1300 22 4636; www.beyondblue.org.au). Participants may also experience some discomfort by wearing an activity monitor on their thigh for one week. However, the monitor is lightweight and will be adhered to the skin by standard post-operative dressing. If any skin irritation occurs, participants will be encouraged to remove it. Participants will also be screened for allergies to adhesive dressing prior to application. In addition, as all participants will be completing a physical assessment of fitness and undertaking exercise as part of the prehabilitation program, there is the risk of the potentially serious event of discomfort and breathlessness that settles quickly when stopping. Participants may also experience short-term fatigue during and immediately after exercise sessions. An unlikely, but potentially serious adverse event is a cardiac arrest or stroke during physical testing or the exercise component of rehabilitation that may create stress on the cardiovascular system. To avoid these risks, participants will be screened for medical precautions and contraindications prior to exercise, assessments will be completed by a trained allied health clinician and a break in assessment or treatment will be completed if a participant needs to stop. This action would most likely settle any symptoms quickly. Participants will be closely monitored for any warning signs or symptoms of compromised cardiac function (chest pain, extreme shortness of breath, dizziness) by a physiotherapist during the exercise intervention and will be working within recommended guidelines. The risk of a serious adverse event is minimised because participants will be screened for medical precautions and contraindications prior to exercise, and patients are only eligible if they have received medical clearance to participate in the trial by their physiotherapist or treating medical practitioner.

In the unlikely event of a serious medical emergency the allied health clinician that is completing the assessment or monitoring the exercise intervention will stop the assessment immediately, assess the situation quickly to ensure safety, send for help by calling 000. If the patient is unresponsive the physiotherapist trained in basic life support will immediately apply basic life support (check airway, check breathing, apply CPR, continue CPR until responsive or normal breathing returns.

There is a potential risk that trial participants could be contacted by unknown “friends” via their Fitbit Dashboard. Within the friend request is a thumbnail picture of the person requesting the friendship. The friend request can be deleted via the Fitbit application. This feature cannot be blocked. This risk will be minimized as he Fitbit dashboard will be setup with separate trial account not directly linked with the participant or social media platforms. Privacy and notification settings within the Fitbit dashboard will be set to the highest possible setting at the commencement of the intervention.

### Handling of Withdrawals

Participants can withdraw from the study by contacting a member of the research team. Data that has already been analysed and/or the results published, may not be able to be withdrawn from the study or destroyed. In such circumstances, data will continue to form part of the study records and study results.

### Replacements

No replacements will be required for participants who chose to withdraw from this study.

# **Statistical Methods**

### Sample Size Estimation & Justification Statistical Methods To Be Undertaken

To gain a preliminary understanding of the effect of the intervention on clinical outcomes, a sample size sufficient to detect a clinically significant between-group difference in physical capacity of 53 m, assuming a large effect size of 1.3 at a power of 0.8 and an alpha level of 0.05, will be sought. It was calculated a sample of 22 patients waitlisted for Auto SCT at Eastern Health would be adequate. No minimal clinically significant difference has been calculated in patients receiving Auto SCT therefore it was estimated to be 41 m based on half a standard deviation^53^ of scores of a mixed cohort of cancer survivors.^54^

Approximately 30 people are treated with ASCT at Eastern Health each year. Our sample size represents a recruitment rate of 75% which is similar to a recently completed cancer rehabilitation trial at Eastern Health. It is anticipated there will be high acceptance of the trial given the lack of existing allied health support for this cohort at Eastern Health. Our scoping study in 2017 highlighted desire from patients to have access to rehabilitation early after cancer diagnosis through programs located onsite at Box Hill Hospital.

*9.2 Statistical Analysis*

The primary outcome (physical capacity at 1 month post-transplant) will be analysed using linear mixed effects models. Modelling will account for variation in baseline values. This method accounts for within-participant dependence of observations over time, and for missing data, allowing some participants to have missing observations at certain time points. If more than 5% of data are missing, a multiple imputation process will be used, providing the assumption data are missing at random is met. A similar approach will be used for analysis of secondary outcomes collected longitudinally. The time spent in moderate to vigorous physical activity will be estimated using a cut-off of 100 steps/minute for moderate intensity physical activity.^27^ The proportion of participants meeting physical activity guidelines will be described. The number of emergency department, SURC presentations and hospital admissions will be reported as an incidence rate ratio using a negative binomial regression model. To avoid bias and to maximize the randomisation process, all available data will be analysed according to allocation (intention to treat analysis), regardless of compliance.

# **Data Security & Handling**

### Details of where records will be kept & How long will they be stored

Electronic data will be stored in a password protected electronic spreadsheet stored in an Eastern Health protected share drive. Hard copy data will be kept in a locked file at the Eastern Health Allied Health Clinical Research Office. At the completion of the study, hard copy data will be kept in a locked archive at Eastern Health for a period of 15 years. All de-identified electronic data will remain in a password protected file on the Eastern Health share drive for a minimum of 15 years before being permanently deleted in accordance with Eastern Health Policy.

###

### Confidentiality and Security

Fitbit Dashboard: An individual study log-in will be set-up via an Outlook account generated specifically for use within the trial. This account will not be linked to the participant and information collected in the Fitbit Dashboard will be de-identified. At the end of the intervention period for each participant, this de-identified data will be downloaded into a csv file and saved within a spreadsheet stored in an Eastern Health protected share drive. Data in the trial account will be deleted immediately after data has been transferred to the Eastern Health sharedrive.

Participant information will be de-identified and allocated an identification number. Lists of names and codes will be kept in a separate file to the data collected so that it is re-identifiable. All hard copy and electronic data will only be able to be accessed by members of the research team. Hard copy data will be kept in the locked file in a locked file at the Eastern Health Allied Health Clinical Research Office and electronic data kept in a password protected spreadsheet in a secure Eastern Health share drive.

### Oversight and Monitoring

The research team for this study will meet regularly. Dr Amy Dennett will be mentored by Prof Nicholas Taylor, who will provide oversight and consultation around the conduct of this study.

# **Appendix**

**List of Attachments included:**

| **Document Name** | **Version Number** | **Date (e.g., 18 January 2012)** |
| --- | --- | --- |
| A: Activity Logbook | 1 | 20/1/20 |
| B: European Organization for Research and Treatment of Cancer QoL Questionnaire Core (EORTC QLQ C30) | 1 | 20/1/20 |
| C: European Organization for Research and Treatment of Cancer QoL Questionnaire High Dose Chemotherapy Module (EORTC QLQ-HDC29) | 1 | 20/1/20 |
| D: Self Efficacy for physical activity questionnaire | 1 | 20/1/20 |
| E: Patient-Generated Subjective Global Assessment | 1 | 20/1/20 |
| F: Flyer | 1 | 20/1/20 |
| G: Cancer Exercise Safety Guide | 1 | 20/1/20 |
| H: Medical Clearance form | 1 | 20/1/20 |
| I: Exercise handout | 1 | 20/1/20 |
| J: Budget | 1 | 20/1/20 |

# **References**

1. Hamadani M. Autologous hematopoietic cell transplantation: An update for clinicians. In*.* Vol 462014:619-632.

2. Majhail NS, Rizzo JD. Surviving the cure: long term followup of hematopoietic cell transplant recipients. *Bone marrow transplantation.* 2013;48(9):1145-1151.

3. Foundation ATABMT. Australasian Bone Marrow Transplant Recipient Registry (ABMTRR). 2020; <https://arrow.org.au/medical-research/australasian-bone-marrow-transplant-recipient-registry-abmtrr/>.

4. Steinberg A, Asher A, Bailey C, Fu JB. The role of physical rehabilitation in stem cell transplantation patients. *Supportive care in cancer : official journal of the Multinational Association of Supportive Care in Cancer.* 2015;23(8):2447-2460.

5. Gielissen MF, Schattenberg AV, Verhagen CA, Rinkes MJ, Bremmers ME, Bleijenberg G. Experience of severe fatigue in long-term survivors of stem cell transplantation. *Bone marrow transplantation.* 2007;39(10):595-603.

6. Syrjala KL, Martin PJ, Lee SJ. Delivering care to long-term adult survivors of hematopoietic cell transplantation. *J Clin Oncol.* 2012;30(30):3746-3751.

7. Fu JB, Lee J, Smith DW, Guo Y, Bruera E. Return to Primary Service Among Bone Marrow Transplant Rehabilitation Inpatients: An Index for Predicting Outcomes. *Archives of physical medicine and rehabilitation.* 2013;94(2):356-361.

8. CAMPBELL KL, WINTERS-STONE KM, WISKEMANN J, et al. Exercise Guidelines for Cancer Survivors: Consensus Statement from International Multidisciplinary Roundtable. 2019;51(11):2375-2390.

9. Jarden M, Baadsgaard MT, Hovgaard DJ, Boesen E, Adamsen L. A randomized trial on the effect of a multimodal intervention on physical capacity, functional performance and quality of life in adult patients undergoing allogeneic SCT. *Bone marrow transplantation.* 2009;43(9):725-737.

10. Liang Y, Zhou M, Wang F, Wu Z. Exercise for physical fitness, fatigue and quality of life of patients undergoing hematopoietic stem cell transplantation: a meta-analysis of randomized controlled trials. *Japanese journal of clinical oncology.* 2018;48(12):1046-1057.

11. van Dongen JM, Persoon S, Jongeneel G, et al. Long-term effectiveness and cost-effectiveness of an 18-week supervised exercise program in patients treated with autologous stem cell transplantation: results from the EXIST study. *Journal of cancer survivorship : research and practice.* 2019;13(4):558-569.

12. Hung YC, Bauer J, Horsley P, Waterhouse M, Bashford J, Isenring E. Changes in nutritional status, body composition, quality of life, and physical activity levels of cancer patients undergoing autologous peripheral blood stem cell transplantation. *Supportive care in cancer : official journal of the Multinational Association of Supportive Care in Cancer.* 2013;21(6):1579-1586.

13. Andersen S, Brown T, Kennedy G, Banks M. Implementation of an evidenced based nutrition support pathway for haematopoietic progenitor cell transplant patients. *Clinical nutrition (Edinburgh, Scotland).* 2015;34(3):536-540.

14. Atkins L, Steer B, Ray H, Kiss N. Implementing and sustaining an evidence-based nutrition service in a haematology unit for autologous stem cell transplant patients. *Supportive Care in Cancer.* 2019;27(3):951-958.

15. Hagiwara S, Mori T, Tuchiya H, et al. Multidisciplinary nutritional support for autologous hematopoietic stem cell transplantation: a cost-benefit analysis. *Nutrition (Burbank, Los Angeles County, Calif).* 2011;27(11-12):1112-1117.

16. Hung YC, Bauer JD, Horsely P, Coll J, Bashford J, Isenring EA. Telephone-delivered nutrition and exercise counselling after auto-SCT: a pilot, randomised controlled trial. *Bone marrow transplantation.* 2014;49(6):786-792.

17. Morishita S, Kaida K, Ikegame K, et al. Impaired physiological function and health-related QOL in patients before hematopoietic stem-cell transplantation. *Supportive care in cancer : official journal of the Multinational Association of Supportive Care in Cancer.* 2012;20(4):821-829.

18. Rotta M, Storer BE, Sahebi F, et al. Long-term outcome of patients with multiple myeloma after autologous hematopoietic cell transplantation and nonmyeloablative allografting. *Blood.* 2009;113(14):3383-3391.

19. Faithfull S, Turner L, Poole K, et al. Prehabilitation for adults diagnosed with cancer: A systematic review of long-term physical function, nutrition and patient-reported outcomes. *European journal of cancer care.* 2019;28(4):e13023.

20. van Haren I, Staal JB, Potting CM, et al. Physical exercise prior to hematopoietic stem cell transplantation: A feasibility study. *Physiotherapy theory and practice.* 2018;34(10):747-756.

21. Schmidt K, Vogt L, Thiel C, Jager E, Banzer W. Validity of the six-minute walk test in cancer patients. *International journal of sports medicine.* 2013;34(7):631-636.

22. ATS statement: guidelines for the six-minute walk test. *Am J Respir Crit Care Med.* 2002;166(1):111-117.

23. van Loo MA, Moseley AM, Bosman JM, de Bie RA, Hassett L. Test-re-test reliability of walking speed, step length and step width measurement after traumatic brain injury: a pilot study. *Brain injury.* 2004;18(10):1041-1048.

24. World Health O. World alliance for patient safety : WHO draft guidelines for adverse event reporting and learning systems : from information to action. In. Geneva: World Health Organization; 2005.

25. Dennett AM, Shields N, Peiris CL, et al. Motivational interviewing added to oncology rehabilitation did not improve moderate-intensity physical activity in cancer survivors: a randomised trial. *Journal of physiotherapy.* 2018;64(4):255-263.

26. Rowe DA, Welk GJ, Heil DP, et al. Stride rate recommendations for moderate-intensity walking. *Med Sci Sports Exerc.* 2011;43(2):312-318.

27. Abel M, Hannon J, Mullineaux D, Beighle A. Determination of step rate thresholds corresponding to physical activity intensity classifications in adults. *Journal of physical activity & health.* 2011;8(1):45-51.

28. Cormie P, Atkinson M, Bucci L, et al. Clinical Oncology Society of Australia position statement on exercise in cancer care. *The Medical journal of Australia.* 2018;209(4):184-187.

29. Skipworth RJ, Stene GB, Dahele M, et al. Patient-focused endpoints in advanced cancer: criterion-based validation of accelerometer-based activity monitoring. *Clinical nutrition (Edinburgh, Scotland).* 2011;30(6):812-821.

30. Taraldsen K, Askim T, Sletvold O, et al. Evaluation of a body-worn sensor system to measure physical activity in older people with impaired function. *Physical therapy.* 2011;91(2):277-285.

31. Grant PM, Dall PM, Mitchell SL, Granat MH. Activity-monitor accuracy in measuring step number and cadence in community-dwelling older adults. *Journal of aging and physical activity.* 2008;16(2):201-214.

32. Kozey-Keadle S, Libertine A, Lyden K, Staudenmayer J, Freedson PS. Validation of wearable monitors for assessing sedentary behavior. *Med Sci Sports Exerc.* 2011;43(8):1561-1567.

33. Maddocks M, Byrne A, Johnson CD, Wilson RH, Fearon KC, Wilcock A. Physical activity level as an outcome measure for use in cancer cachexia trials: a feasibility study. *Supportive care in cancer : official journal of the Multinational Association of Supportive Care in Cancer.* 2010;18(12):1539-1544.

34. Dennett AM, Peiris CL, Shields N, Prendergast LA, Taylor NF. Cancer Survivors Awaiting Rehabilitation Rarely Meet Recommended Physical Activity Levels: An Observational Study. *Rehabilitation Oncology.* 2018;36(4):214-222.

35. Masse LC, Fuemmeler BF, Anderson CB, et al. Accelerometer data reduction: a comparison of four reduction algorithms on select outcome variables. *Med Sci Sports Exerc.* 2005;37(11 Suppl):S544-554.

36. Gleeson M, Bishop NC, Stensel DJ, Lindley MR, Mastana SS, Nimmo MA. The anti-inflammatory effects of exercise: mechanisms and implications for the prevention and treatment of disease. *Nature reviews Immunology.* 2011;11(9):607-615.

37. Seruga B, Zhang H, Bernstein LJ, Tannock IF. Cytokines and their relationship to the symptoms and outcome of cancer. *Nature reviews Cancer.* 2008;8(11):887-899.

38. Rogers LQ, Vicari S, Trammell R, et al. Biobehavioral factors mediate exercise effects on fatigue in breast cancer survivors. *Med Sci Sports Exerc.* 2014;46(6):1077-1088.

39. Luckett T, King MT, Butow PN, et al. Choosing between the EORTC QLQ-C30 and FACT-G for measuring health-related quality of life in cancer clinical research: issues, evidence and recommendations. *Annals of oncology : official journal of the European Society for Medical Oncology.* 2011;22(10):2179-2190.

40. Uwer L, Rotonda C, Guillemin F, et al. Responsiveness of EORTC QLQ-C30, QLQ-CR38 and FACT-C quality of life questionnaires in patients with colorectal cancer. *Health and quality of life outcomes.* 2011;9:70.

41. Velikova G, Weis J, Hjermstad MJ, et al. The EORTC QLQ-HDC29: a supplementary module assessing the quality of life during and after high-dose chemotherapy and stem cell transplantation. *Eur J Cancer.* 2007;43(1):87-94.

42. Schwarzer R, Lippke S, Luszczynska A. Mechanisms of health behavior change in persons with chronic illness or disability: the Health Action Process Approach (HAPA). *Rehabil Psychol.* 2011;56(3):161-170.

43. Schwarzer R. Modeling Health Behavior Change: How to Predict and Modify the Adoption and Maintenance of Health Behaviors. 2008;57(1):1-29.

44. Ungar N, Wiskemann J, Sieverding M. Physical Activity Enjoyment and Self-Efficacy As Predictors of Cancer Patients' Physical Activity Level. *Frontiers in Psychology.* 2016;7:898.

45. Jager-Wittenaar H, Ottery FD. Assessing nutritional status in cancer: role of the Patient-Generated Subjective Global Assessment. *Current opinion in clinical nutrition and metabolic care.* 2017;20(5):322-329.

46. Bauer J, Capra S, Ferguson M. Use of the scored Patient-Generated Subjective Global Assessment (PG-SGA) as a nutrition assessment tool in patients with cancer. *European journal of clinical nutrition.* 2002;56(8):779-785.

47. Roberts HC, Denison HJ, Martin HJ, et al. A review of the measurement of grip strength in clinical and epidemiological studies: towards a standardised approach. *Age and ageing.* 2011;40(4):423-429.

48. RW. B. Dynamometer measurements of hand-grip strength predict multiple outcomes. *Percept Mot Skills.* 2001;93(2):323-328.

49. Sasaki H KF, Yamada M, Fujita S. Grip strength predicts cause-specific mortality in middle-aged and elderly persons. *Am J Med* 2007;120(4):337-342.

50. Guerra RS, Fonseca I, Pichel F, Restivo MT, Amaral TF. Handgrip strength cutoff values for undernutrition screening at hospital admission. *European journal of clinical nutrition.* 2014;68(12):1315-1321.

51. Ozorio GA, Barao K, Forones NM. Cachexia Stage, Patient-Generated Subjective Global Assessment, Phase Angle, and Handgrip Strength in Patients with Gastrointestinal Cancer. *Nutrition and cancer.* 2017;69(5):772-779.

52. Guerra RS, Amaral TF, Sousa AS, et al. Handgrip strength measurement as a predictor of hospitalization costs. *European journal of clinical nutrition.* 2015;69(2):187-192.

53. Norman GR, Sloan JA, Wyrwich KW. Interpretation of Changes in Health-Related Quality of Life: The Remarkable Universality of Half a Standard Deviation. *Medical Care.* 2003;41(5):582-592.

54. Schmidt K, Vogt L, Thiel C, Jäger E, Banzer W. Validity of the Six-Minute Walk Test in Cancer Patients. *International journal of sports medicine.* 2013;34(07):631-636.

**Appendix A: Activity Logbook**

**ACTIVITY LOG BOOK**

**NAME: ______________________________**

**Time/date on:** __________________________ Time/date off: _________________

| **DAY** | **LIST MAIN ACTIVITIES COMPLETED & APPROXIMATE TIME SPENT**  For example, watched television (1 hour), went for a walk (30 mins) | **PROBLEMS**  **(include any time the activity monitor was taken off and why?)** |
| --- | --- | --- |
| 1  Day: (e.g. Saturday) |  |  |
| 2  Day: |  |  |
| 3  Day: |  |  |
| 4  Day: |  |  |
| 5  Day: |  |  |
| 6  Day: |  |  |
| 7  Day: |  |  |

**Appendix B: EORTC-C30**

[**https://www.eortc.org/app/uploads/sites/2/2018/08/Specimen-QLQ-C30-English.pdf**](https://www.eortc.org/app/uploads/sites/2/2018/08/Specimen-QLQ-C30-English.pdf)

**Appendix C: BMT EORTC-HDC 29**

[**https://qol.eortc.org/questionnaires/**](https://qol.eortc.org/questionnaires/)

**Appendix D:**

**Self-efficacy for physical activity**

Please answer the following statement in relation to the circumstances below. Please tick the best answer for you.

Are you confident that you can permanently be regularly physically active?

| ***“I am confident that I can permanently be regularly physically active…”*** | | | | |
| --- | --- | --- | --- | --- |
| … even if I have side-effects (e.g. nausea) of the cancer therapy | Not at all | Partially agree | Mostly agree | Totally agree |
| … even if I am tired | Not at all | Partially agree | Mostly agree | Totally agree |
| … even if you have pain when exercising | Not at all | Partially agree | Mostly agree | Totally agree |
| … even if you were too busy with other activities or appointments | Not at all | Partially agree | Mostly agree | Totally agree |
| … even if you had to exercise alone | Not at all | Partially agree | Mostly agree | Totally agree |
| … even if you felt stressed | Not at all | Partially agree | Mostly agree | Totally agree |
| … even if you felt depressed | Not at all | Partially agree | Mostly agree | Totally agree |

**Appendix E: Patient-Generated Subjective Global Assessment**

[**https://pt-global.org/pt-global/**](https://pt-global.org/pt-global/)


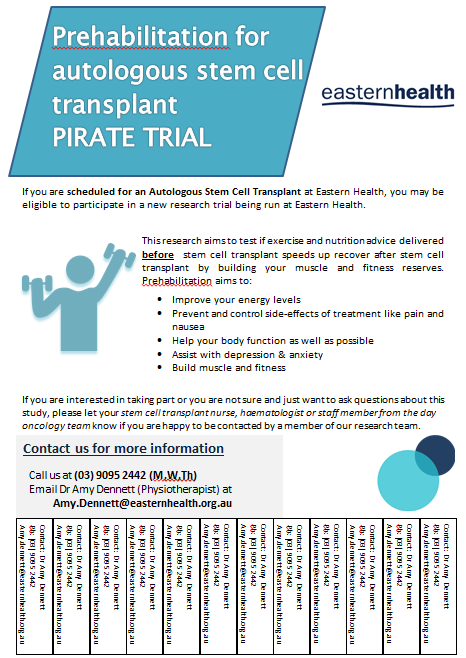
**Appendix F: Flyer**

**Appendix G:**

**Safety Reference Guide for Exercise for People with Cancer**

*Reference: Santa Mina DS, Langelier D, Adams SC, Alibhai SMH, Chasen M, Campbell KL, Oh P, Jones JM, Chang E. Exercise as part of routine cancer care. Lancet Oncol. 2018 Sep;19(9):e433-e436*

**Absolute Contra-indications**

- unstable angina, arrhythmia, hypertension or heart failure
- acute embolus/infarct, acute systemic infection with fever
- dissecting aneurysm
- acute myocarditis or pericarditis

Discontinue exercise if there are adverse cardiorespiratory signs or symptoms: moderately severe angina, dizziness or pre-syncope, cyanosis or pallor – Call Code Blue

| **Relative Contra-indication**  **Gain physician clearance** | **Exercise modification** |
| --- | --- |
| **Platelets**  <20 × 10^9^/L | - Functional mobility exercises only - Minimise fall or impact risk - Emphasise normal breathing (avoid Valsalva manoeuvre) monitor bruising and bleeding |
| **Haemoglobin**  <80 g/L | - Low to moderate intensity only |
| **White blood cell**  <2·0 × 109/L: | - Avoid group exercise - Light to moderate intensity exercise only - Reinforce importance of sanitisation and hand-washing Clean all equipment before use |
| **Neutrophils**  <1·5 × 109/L | - Avoid group exercise - Light to moderate intensity exercise only - Reinforce importance of sanitisation and hand-washing Clean all equipment before use |
| **Blood glucose**  <5·5 mmol/L | - Recommend 5–30 g carbohydrate consumption before exercise |
| **Resting Blood pressure**  Systolic blood pressure >200 mmHg or diastolic blood pressure >110 mmHg after two measurements, 5 min apart  Diastolic blood pressure 90–110 mmHg after  two measurements, 5 min apart | - Monitor for signs and symptoms - Repeat blood pressure measurements frequently during session |
| **Resting heart rate**  <60 bpm after two measurements, 5 min apart  >120 bpm after two measurements, 5 min | - Exercise as tolerated - Monitor closely |
| **Resting O2 saturation**  ≤88% | - Discontinue exercise if desaturation to this level occurs during a session - 88-94% Exercise as tolerated, monitor signs and symptoms of fatigue and exertion, repeat SpO_2_ |
| **Sudden or severe pain, swelling or dysfunction** | - Avoid exercise of the affected region - Doctor’s clearance required to resume exercise of the affected region or if general exercise exacerbates symptoms |

# **Appendix H: Medical Clearance Form**

Eastern Health Prehabilitation for Autologous Stem Cell Transplant

To Dr _____________

Re:

_________has enrolled into the Prehabilitation for Autologous Stem Cell Transplant Trial. Physiotherapy screening has identified a concern that may impact on their ability to exercise safely ______________________________________________

Please tick one or more boxes

__________is medically fit to participate in exercise including:

- aerobic exercise including treadmill walking and recliner bike
- strength / weight training for upper and lower body
- balance training
- stretches

Special Precautions: __________________________________________________

___________________________________________________________________

___________________________________________________________________

Other: ______________________________________________________________

___________________________________________________________________

***Please attach any recent discharge summaries and*** ***fax to 9955 1388***

| Doctors Signature: | Date: |
| --- | --- |
| Doctor’s Name (printed): | Phone No: |

If you have any queries, please don’t hesitate to contact Amy Dennett via email: **amy.dennett@easternhealth.org.au** or phone 9095 2442

**Appendix I: Exercise handout**

<https://www.exerciseismedicine.org/eim-in-action/moving-through-cancer/>

- “[Being Active When You Have Cancer](https://www.exerciseismedicine.org/wp-content/uploads/2021/04/EIM_Rx-for-Health_Cancer.pdf)” patient handout
- “[Sit Less. Move More.](https://www.exerciseismedicine.org/wp-content/uploads/2021/04/EIM_Rx-for-Health_Sit-Less-Move-More.pdf)” patient handout

**Appendix J: Budget**

| *Human resources* | | | | |
| --- | --- | --- | --- | --- |
| Physiotherapist  0.3 EFT 12 hours/ week, 10 months  Dietitian  0.1 EFT 4 hours/week, 9 months | | Oversee the project including participant recruitment, organisation of appointments and management of data as well as data collection and assisting with the prehabilitation intervention  To deliver nutrition intervention | | **$25,231         $7,570** |
| *Maintenance/consumables* | | | | |
| *Blood Assays*  *Accelerometers* | A blood analysis is required to measure levels of systemic inflammation with the biomarkers C-reactive protein, $350 setup fee, and $9.70 x 3 occasions x 22 participants | | **$932**  ***In kind*** | |
| *Car Parking* | | | | |
|  | | 18 visits x 11 patients x $10/day  3 visits x 11 patients x $10/day | | **$1,980**  **$330** |
| **TOTAL $36,043** | | | | |
